# Impact of *Acacia*-derived biochar to mitigate salinity stress in *Zea mays* L. by morpho-physiological and biochemical indices

**DOI:** 10.1038/s41598-024-83010-5

**Published:** 2024-12-30

**Authors:** Ghulam Murtaza, Gang Deng, Muhammad Usman, Arslan Jamil, Muhammad Qasim, Javed Iqbal, Sezai Ercisli, M. Irfan Akram, Muhammad Rizwan, Mohamed S. Elshikh, Humaira Rizwana, Zeeshan Ahmed, Rashid Iqbal

**Affiliations:** 1 School of Agriculture, Yunnan University, Kunming, 650504 Yunnan China; 2 School of Ecology and Environmental Sciences, Biocontrol Engineering Research Center of Crop Diseases and Pests, Yunnan University, Kunming, 650500 Yunnan Province China; 3 School of Agriculture and Biology, Shanghai Jiao Tong University, 800 Dongchuan Road, Minghang District, Shanghai, 200240 China; 4 Yunnan Key Laboratory of Green Prevention and Control of Agricultural Transboundary Pests, Agricultural Environment and Resources Institute, Yunnan Academy of Agricultural Sciences, Kunming, 650205 Yunnan China; 5 Microelement Research Center, College of Resources and Environment, Huazhong Agricultural University, Wuhan, 430070 Hubei China; 6 Department of Botany, Bacha Khan University, Charsadda, 24420 Khyber Pakhtunkhwa Pakistan; 7 Department of Horticulture, Agricultural Faculty, Ataturk University, 25240 Erzurum, Turkey; 8 Department of Entomology, Faculty of Agriculture and Environment, The Islamia University of Bahawalpur, Bahawalpur, 63000 Pakistan; 9 Institute of Crop Science and Resource Conservation (INRES), University of Bonn, 53115 Bonn, Germany; 10 Department of Botany and Microbiology, College of Science, King Saud University, Riyadh, 11451 Saudi Arabia; 11 Xinjiang Institute of Ecology and Geography, Chinese Academy of Sciences, Urumqi, 830011 Xinjiang China; 12 Xinjiang Institute of Ecology and Geography, Cele National Station of Observation and Research for Desert-Grassland Ecosystems, Chinese Academy of Sciences, Xinjiang, 848300 China; 13 College of Life Science, Shenyang Normal University, Shenyang, 110034 China; 14 Department of Agronomy, Faculty of Agriculture and Environment, The Islamia University of Bahawalpur, Bahawalpur, 63100 Pakistan; 15 Department of Life Sciences, Western Caspian University, Baku, Azerbaijan

**Keywords:** Biochar, Salinity, Growth, Physiology, Soil, Antioxidant activities, Acacia, Corn, Biochemistry, Plant sciences

## Abstract

Climate change has caused many challenges to soil ecosystems, including soil salinity. Consequently, many strategies are advised to mitigate this issue. In this context, biochar is acknowledged as a useful addition that can alleviate the detrimental impacts of salt stress on plants. The objective of this study is to evaluate the effects of different levels of salt (Control; T0 0 gl^−1^, T1; 1.50, and T2; 3 gl^−1^) and biochar addition rates (A0; 0 g kg^−1^, A1; 40 g kg^−1^, and A2; 80 g kg^−1^) on the agronomic, physiological, and biochemical responses of corn plants. The results of our study showed a significant increase in the biomass of corn plants when exposed to salt stress and treated with 40 g kg^−1^ of biochar. The result underscores the significant function of *Acacia*-biochar in mitigating salt toxicity. The application of A1 biochar at a specified rate mitigated the adverse effects of salt-induced oxidative stress by augmenting the activities of catalase (CAT) and glutathione-S-transferase (GST). Furthermore, the utilization of biochar led to an increase in chlorophyll b concentrations in maize plants subjected to saline water treatment. Biochar is generally considered an efficient method for alleviating the adverse effects of salinity. To enhance plant growth and development while mitigating salinity-induced toxicity, the application of biochar in saline soils must be implemented appropriately.

## Introduction

Crop productivity is significantly impacted by abiotic stressors, including soil salinity^1^. Conversely, over 800 million hectares of land globally are impacted by salt stress^2^. Agricultural soils experience degradation from salinity mostly because of irrigation with polluted water, biosolids, phosphate fertilizers, and sewage sludge^3^. Salinity stress has been proven to reduce plant biomass, growth, and uptake of mineral nutrients^4^. Likewise, salt stress reduced calcium and potassium levels in maize seedlings. Salinity-induced oxidative stress in maize decreased the activity of antioxidant enzymes^5^.

Maize (*Zea mays* L.) is a crucial component of the human diet and serves as the primary food source for the majority of the global population. In 2020, maize production in Asia was approximately 318 MT, while global production reached over 713 MT. In 2020, Pakistan produced approximately 24.23 million tons of maize from an area of 8.69 million hectares, positioning the country among the top 10 wheat producers globally^6^. Furthermore, maize may thrive in marginal soils to sustain the burgeoning population and is irrigated with sewage water because of the scarcity of high-quality irrigation sources^7^. In actual field situations, soils primarily exposed to sewage sludge or wastewater frequently endure several stressors, including salt^8^. Saline soils have low fertility and possess a high bioavailability of toxic metals^9^. Soil salinity concurrently exerts detrimental impacts on plants, beyond the impact of these stresses individually^10^. The administration of NaCl reduced plant growth, chlorophyll levels, and RWC^11^. NaCl stress reduced the height of the plant, length of roots, MDA level, and antioxidant enzyme activity in wheat^11^. NaCl stress on the leaves enhanced metal accumulation in wheat relative to the control^8^. The stress of NaCl elevated the MDA levels in maize seedlings relative to control^9^.

Various techniques have been established to mitigate the detrimental effects of salinity stress in crops^10^. Using biochar an organic substance subjected to pyrolysis in an oxygen-limited environment, as a soil amendment is attracting significant global interest^12^. Several researches have documented the beneficial impacts of BC under conditions of salinity^8^. The incorporation of biochar into the soil elevated the soil pH and reduced the uptake and bioavailability by crops^12^. Biochar is more efficient in decreasing salinity impacts than other organic amendments^13^. Likewise, biochar administration enhanced potato growth, yield, and photosynthesis under saline stress, while reducing sodium ions and elevating potassium ions levels in the xylem^14^. The combination of biochar with plant growth-promoting bacteria enhanced maize growth and biomass, reduced sodium ion levels, and elevated potassium ion concentration in the xylem sap of maize^12^. The incorporation of biochar mitigated oxidative stress and enhanced bean development in conditions of salt stress^10^. There is less evidence of the impact of the wood biochar amendment on maize cultivated in saline soil. We anticipated that biochar could mitigate saline stress in maize. The objective of this study was to examine the impact of *Acacia*-derived Biochar to Mitigate Salinity Stress in *Zea mays* L. by Morpho-physiological and Biochemical Indices.

## Materials and methods

### Site, plant and growth environment description

The study was conducted at the agricultural research centre located at Bahawalpur (29.3544° N, 71.6911° E) in Pakistan. To do this, a sample of soil measuring 15 cm to 20 cm was taken from an area located in Islamia University of Bahawalpur, air-dried overnight, homogenized, and sieved via a 2 mm sieve. Subsequently, the soil was put into plastic pots measuring 40 cm in diameter, with each pot containing 1 kg of soil. The soil was then subjected to a completely random design, involving two Parameters: salinity treatments (Control; T0 gl^−1^, T1; 1.50, and T2; 3 gl^−1^) and different doses of biochar (A0; 0 g kg^−1^, A1; 40 g kg^−1^, and A2; 80 g kg^−1^). The study consisted of a total of nine treatments, conducted using a completely randomized experimental design with five replicas for each treatment/application. The biochar utilized was derived from *Acacia* bark (*Acacia* bark taken from Regional Agricultural Research Institute Bahawalpur) and synthesized through pyrolysis at a temperature of 500 °C for 4 h. The physicochemical characteristics of biochar are described in Table 1. The salinity of the soil water was established by utilizing NaCl and DI water. In Petri dishes, the seeds of maize (variety-SILVER-2019, taken from Regional Agricultural Research Institute Bahawalpur) were pre-soaked on wet paper for 24 h at a temperature of 25 °C. Plastic pots were utilized to cultivate maize plants, with each pot containing 5 seeds. The pots were filled with 1 kg of sandy clay soil and 150 g of gravel. The plastic pots were kept to control climate conditions within a greenhouse for duration of 90 days, specifically from February to April. The average lowest and highest temperatures for this period ranged from 19 °C to 28 °C, while the photoperiod was maintained at 14 h. Additionally, manual irrigation was conducted at a level equivalent to 90% of the soil’s holding capacity to be capable of leaching for the duration of the application period.

**Table 1:** Key physical and chemical characteristics of *Acacia*-biochar.

Characteristic	Acacia-biochar
pH	9.6
CEC (mmol_c_kg^−1^)	271
EC (µS cm^−1^)	413
SSA (m^2^ g^−1^)	35.303
C (%)	70.18
H (%)	4.13
N (%)	0.76
O (%)	20.56
O/C	0.22
H/C	0.71
Mg (g kg DM)	0.99
Ca (g kg DM)	14.02
K (g kg DM)	3.19
Na (g kg DM)	1.0
Fe (g kg DM)	1.0
P (g kg DM)	0.29
Cl (g kg DM)	311
Zn (g kg DM)	5
SO_4_ (g kg)	119
NO_3_ (g kg DM)	2.6
Mineral matter (g kg)	280
Organic matter level (g kg)	719

### Analysis of plants

#### Growth related parameters

After the harvest, the corn plants were separated into roots and shoots and then underwent thorough washing using distilled water. A total of 100 g of each plant part were put in a hot air oven at a temperature of 65 °C for 40 h. After that, the percentage of dry matter was determined using the methodology described by Helaoui et al.^15^.

1 $$\:DM\:\%=(Fresh\:matter/Dry\:matter)\times\:100$$

Upon that, the length of the root and shoot, as well as the fresh weight of the corn plant, were measured and then stored at a temperature of -80 °C until the chlorophyll determination and biochemical analysis were conducted.

#### Chlorophyll determination

The determination of total chlorophyll concentrations was conducted following the methodology described by Pan et al.^16^. In this experiment, a volume of 20 ml of 80% acetone was administrated to 1 gram of fresh leaves. After that, the mixture underwent filtration, prompting the measurement of the optical density of Chlorophyll a and b at wavelengths of 663 nm and 645 nm, respectively. The determination of pigment contents was conducted by applying the protocol stated by Arnon^17^.

#### Enzyme determination

Maize fresh roots and shoots (0.5 gram) were mixed with 1 ml of phosphate buffer (pH 7.5; 100 ml DI water; 30 mM MOPS; 5 mM Na_2_-EDTA; 10 mM DTT; 10 ascorbic acid, PVP 0.6%), 10 µl of the ascorbic acid (1 M), and Crospovidone 10 mg. The solution underwent centrifugation with a force of 15,000 g for 10 min at a temperature of 4 °C. After that, the protein content was assessed by the Bradford method^18^, employing bovine serum albumin as the reference standard. The supernatant was applied to determine GST and CAT enzyme activities. The activity of CAT was evaluated using the Aebi et al.^19^ technique, which involved monitoring the absorbance at 240 nm to measure the decomposition of hydrogen peroxide. To do this, a volume of 20 µl of enzyme extract was mixed into a solution containing 780 µl of phosphate buffer (pH 7.5) and 200 µl of hydrogen peroxide. The activity of CAT was quantified in units of µmol min^−1^ mg^−1^ protein. The analysis of GST activity was conducted in accordance with Habig et al.^20^. The sample mixture consisted of 1 ml of phosphate buffer with a pH of 7.5, 50 µl of glutathione with a concentration of 4 mM, CDNB 50 µl, and enzyme extract 50 µl. The activity of GST was assessed by employing an extinction coefficient of 9.6 mM/cm through kinetic measurement at a wavelength of 340 nm. The findings were quantified in units of mol min^1^ mg^−1^ of protein.

#### Maize plants’ MDA content

The analysis of lipid peroxidation in corn plants after a 90-day culture period was conducted using the colorimetric approach developed by Sytykiewicz et al.^21^. In this experiment, 0.3 g of frozen materials were mixed into a 3 ml solution containing 15% Trichloroacetic acid (15%), hydrochloric acid (0.25 M; 0.01%), and Thiobarbituric acids (0.37%). After that, the samples were subjected to incubation at a temperature of 95 °C for 30 min. The homogenates underwent centrifugation at a force of 12,000 g for duration of 15 min. The resulting solution was then subjected to absorbance measurements at wavelengths of 532 nm and 600 nm. The findings were presented in the form of nmol g^−1^ fresh weight.

### Statistical analysis

Statistical analysis was conducted with SPSS Software (V-21). The distribution’s normality was confirmed using the Shapiro-Wilk test. The application of two-way analysis of variance (ANOVA) was used to assess significant differences among treatments (*p* < 0.05).

## Results

### Biochar application effects on plants length

**Fig. 1:**
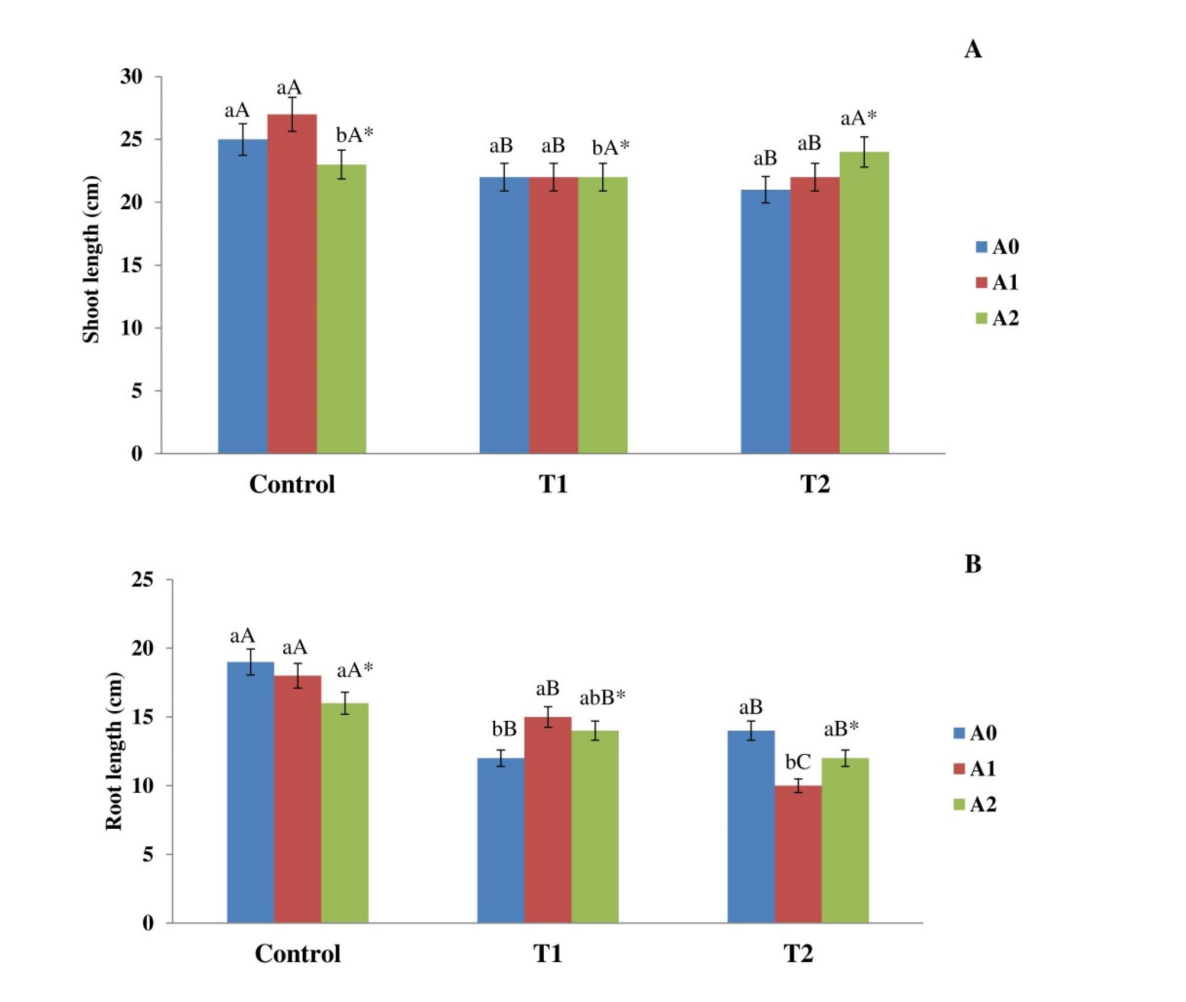
Impact of salt levels (Control, T1, and T2) and biochar dose (A0, A1, and A2) on the length of maize plant shoots (**A**) and roots (**B**) following a 90-day exposure period. The means, denoted by various capital letters, exhibited a significant disparity for the same biochar level across the various treatments. The means, shown by various lowercase letters, exhibited a notable disparity in biochar addition rates within the same application. *The interaction between salt level and biochar application rate has a significant impact.

Figure 1 elucidates the observed variations in the corn plant length after co-exposure to salinity and biochar addition/treatment. The findings indicated a significant correlation between salt levels and biochar rates in both T1 and T2. The effect of biochar is contingent upon the concentration of salt. In the absence of biochar, the shoots subjected to T1 and T2 exhibited a notable decrease in length, with average measurements of 22 ± 0.29 cm and 22.12 ± 1.3 cm, respectively, in contrast to the control treatment value of 24.9 ± 0.4 cm. The inclusion of A1 led to an enhancement in shoot length in the control group relative to the treatment without biochar. The recorded values were 28.1 ± 1.19 cm. Nevertheless, those variations were not found statistically significant. Incorporating A2 increased the lengths of shoots in T1 and T2 plants, the values reached 23 ± 1.9 cm and 24.19 ± 2.01 cm, respectively, in comparison to the treatment without the addition of biochar (Fig. 1A). Nonetheless, this increase was not significant. The maize plants subjected to T1 and T2 demonstrated a reduction in root length relative to the control group, recording lengths of 13 ± 0.02 cm and 14.15 ± 1.03 cm, respectively, in contrast to the control measurement of 20.71 ± 1.2 cm (Fig. 1B). The inclusion of A1 led to a substantial increase in the root length of maize plants subjected to T1 treatment, in contrast to the treatment without biochar. The length percentage increase observed was 19%. Nevertheless, A1 experienced a significant reduction in root length as compared to T2. This shows the positive correlation between the biochar addition rate and salt level.

### Effect biochar addition on the weight of plants

**Fig. 2:**
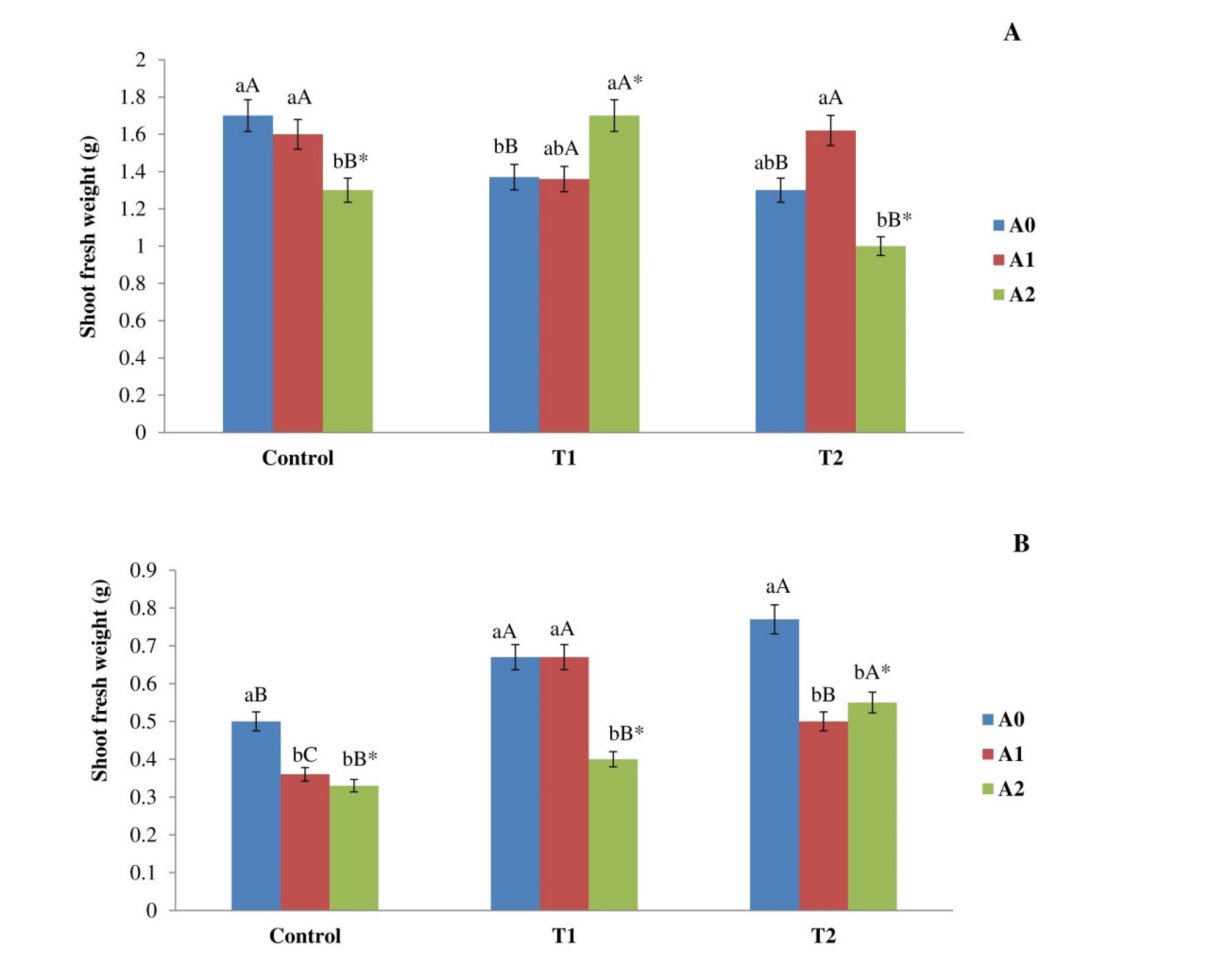
Impact of salt levels (Control, T1, and T2) and rate of biochar (A0, A1, and A2) on the weight (g) of (**A**) shoots and (**B**) roots of corn plants after a 90-day exposure period. The means, denoted by various capital letters, exhibited a significant disparity for the same rate of biochar across the diverse treatments. The means, shown by various lowercase letters, exhibited a significant disparity in biochar application rates within the same treatment. The interaction between biochar rate and salinity has a *significant impact.

Figure 2 illustrates the impact of salt level on the weight of corn plant shoots and roots following a 90-day exposure period, both with and without biochar. The salt stress resulted in a significant decrease in shoot weight in T1 and T2 plants when compared to the control group. The measured values were 1.35 ± 0.06 g and 1.38 ± 0.116 g, respectively, while the control group had a shoot weight of 1.70 ± 0.06 g. Moreover, a substantial impact was noted due to the correlation between saline levels and the rate of biochar application. Nonetheless, the inclusion of T1 led to a substantial increase in the shoot weight of maize plants subjected to T1 and T2 treatments, in contrast to the treatment devoid of biochar, with length enhancements of 13% and 18%, respectively. Furthermore, the application of T2 resulted in an increase in the shoot weight of corn plants subjected to T1 treatment, as compared to the treatment without biochar. The percentage of length augmentation observed was 20%. Nevertheless, the incorporation of T2 resulted in a reduction in shoot weight subjected to T2 treatments compared to treatment without biochar, with a reduction of 25% (Fig. 2A). In the absence of biochar (A0), the root weight in T1 and T2 plants was significantly higher than that of the control treatment. The root weight readings were 0.67 ± 0.04 g and 0.77 ± 0.04 g, respectively, in comparison to a control value of 0.5 ± 0.001 g. The inclusion of A1 led to a decrease in the root weight of maize plants exposed to T2 and control treatments, relative to treatments lacking biochar. The corresponding reduction percentages were 29% and 35%. The injection of A2 led to a decrease in root weight in plants subjected to T1, T2, and control treatments compared to those without biochar, with reductions of 40%, 29%, and 35%, respectively (Fig. 2B).

### Effect of biochar application on dry matter of plants

**Fig. 3:**
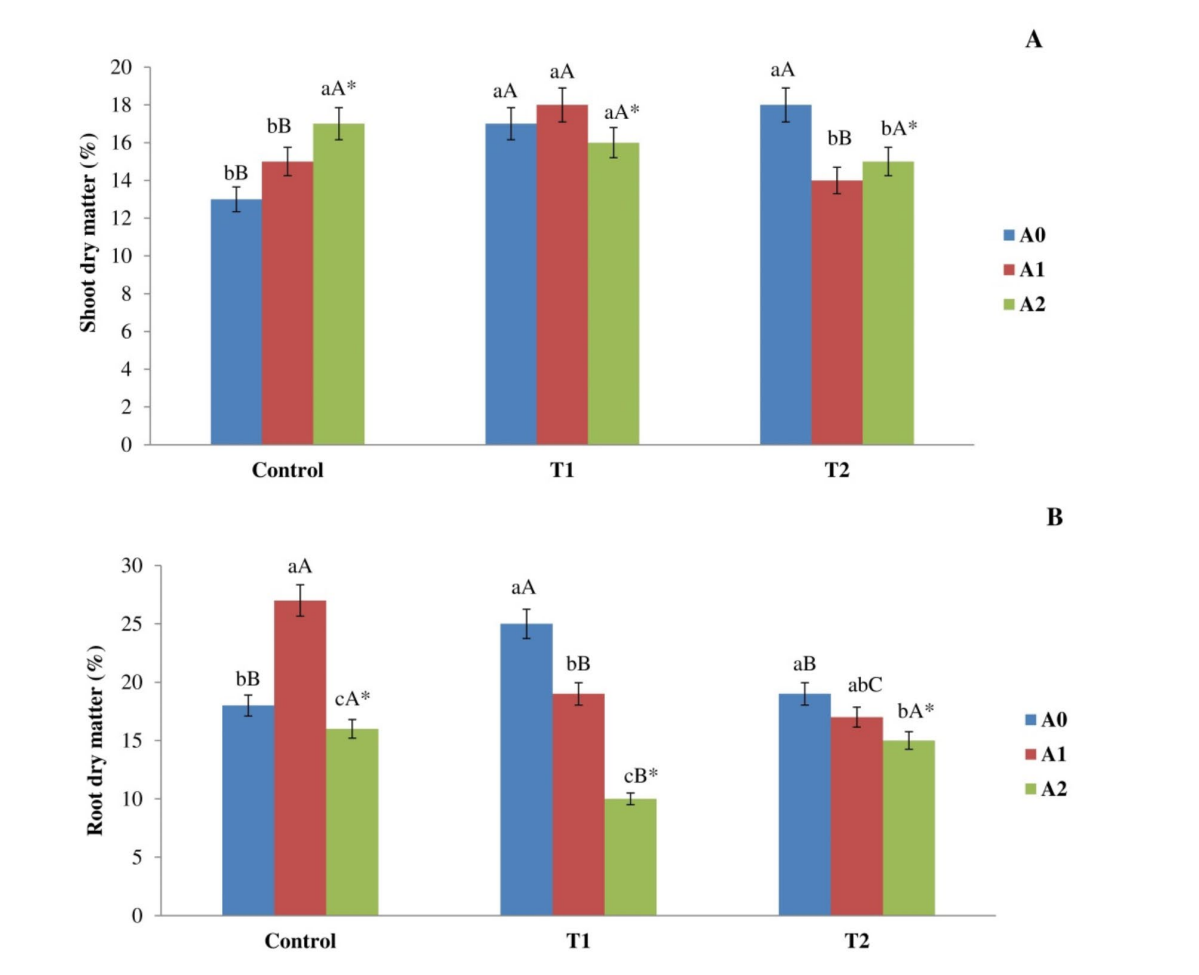
Impact of salt level (Control, T1, and T2) and biochar rate (A0, A1, and A2) on the percentage of dry matter in corn plant (**A**) shoots (**B**) roots was assessed following exposure of 90 days. The methods employed by various capital letters shown a significant disparity in the same rate of biochar addition across various treatments. The methodologies employed by various lowercase letters showed a significant disparity in biochar rates within the identical treatment. *: The interaction between biochar rate and salinity has a significant impact.

After exposure of 90 days, the T1 and T2 treatments showed a significant rise in the dry matter of the shoots than exposed to control soils (Fig. 3A). The values were 17.02 ± 1.02% and 18.6 ± 0.72%, whereas the control value was 13.07 ± 1.04%. The relationship between salt concentration and biochar application rate significantly influenced the plant’s response, which is dependent on the salt level. Control shoots subjected to A1 had a notable increase in dry matter relative to the lack of biochar treatment, culminating in a 12% enhancement. Nevertheless, there was a significant reduction in the amount of dry matter in T2 shoots that were treated with A1, as compared to the treatment absence of biochar. The reduction in dry matter percentage was measured at 15%. Subsequently, in the roots and absence of biochar, our findings indicated a significant rise in T1 treatments, with values of 27.77 ± 1.02%, in comparison to the control group value of 20.19 ± 0.60% respectively. Upon administering treatment A1, we noted a substantial increase in the plants cultivated in the control group, as opposed to the roots without biochar supplementation, with a 34% elevation in the recorded values. However, the application of the same quantity of biochar led to a notable decrease in plants subjected to T1 compared to those without biochar, yielding a reduction of 30%. Moreover, treatment with A2 resulted in a notable decrease in the dry matter of roots when compared to the control, T1, and T2 treatments, with reductions of 13%, 59%, and 16%, respectively, in the absence of biochar (Fig. 3B).

### Application of biochar impacted the measurement of chlorophyll

**Fig. 4:**
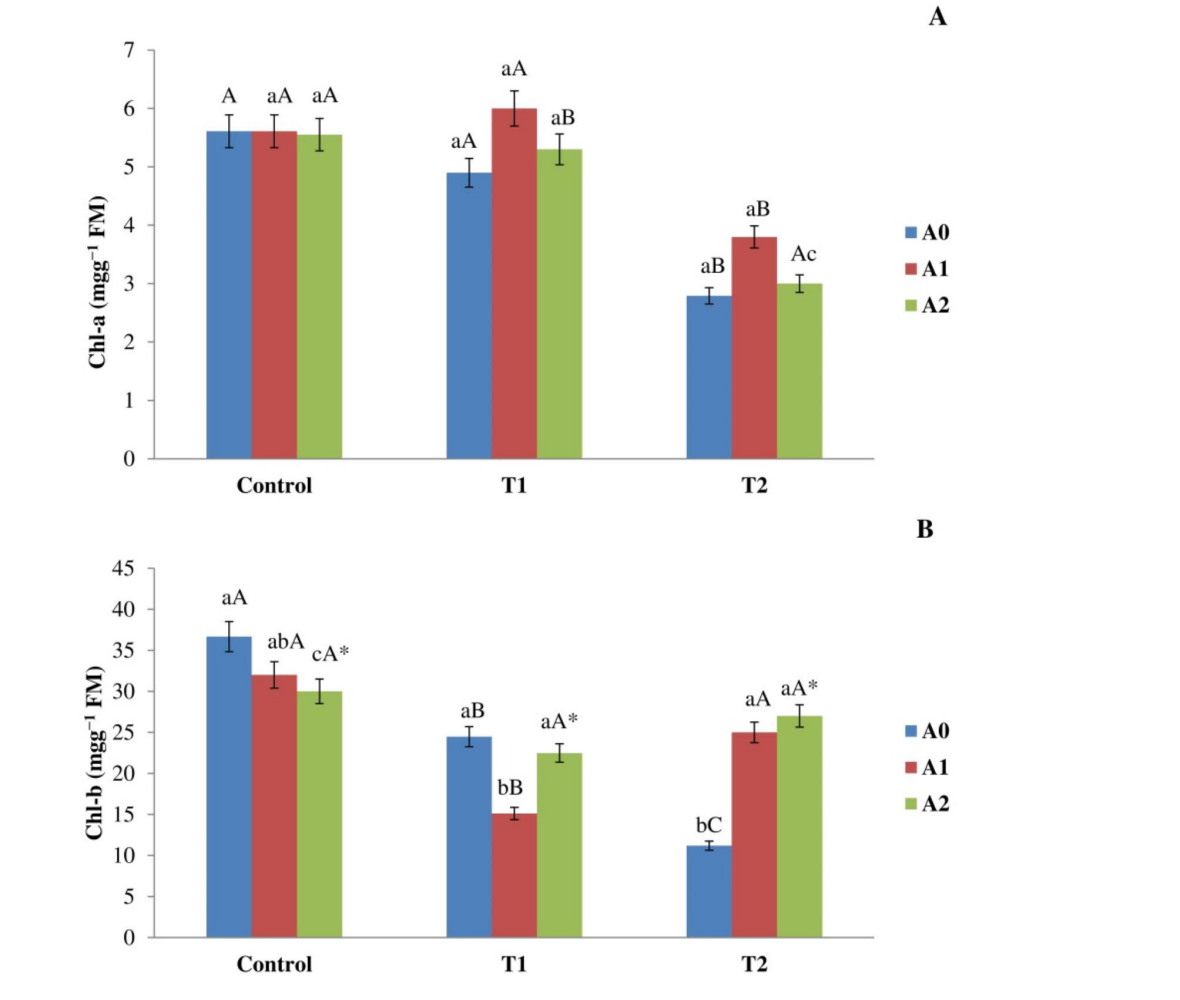
Impact of salt level (Control, T1, and T2) and biochar rate (A0, A1, and A2) on (**A**) Chlorophyll a and (**B**) Chlorophyll b levels in maize leaves following a 90-day exposure period. The means employed by various capital letters show a significant disparity in the same biochar addition rate across various treatments. The means employed by various lowercase letters revealed a significant disparity in biochar addition rates within the same treatment. *The interactions between biochar rate and salinity level had a significant impact.

Salt stress decreased the chlorophyll content of maize plants, as illustrated in Fig. 4. The level of chlorophyll a markedly diminished in leaves subjected to T2, yielding a value of 2.79 ± 0.17 mgg^−1^ FM, in contrast to the control group’s value of 5.61 ± 0.37 mgg ^−1^ FM. Moreover, the relationship between the biochar application rate and salinity levels did not have any significant effect on Chlorophyll (a) There was no significant disparity detected in maize plants treated with A1 and A2 compared to plants that were not treated with biochar. Nevertheless, both the biochar rate and salt level had significant effects on the Chlorophyll (b) The latter value reduced from 36.67 ± 2.69 mgg^−1^ FM in control leaves to 24.47 ± 2.21 mgg^−1^ FM and 11.19 ± 1.88 mgg^−1^ FM in plants treated to T1 and T2, respectively. The inclusion of A1 led to a notable fall in Chlorophyll b levels, especially in leaves treated with T1, in contrast to plants lacking biochar, exhibiting a reduction of 28%. Nonetheless, the incorporation of A1 and A2 markedly elevated the concentrations of Chlorophyll b in T2 leaves relative to those devoid of biochar, with enhancements of 63% and 65% respectively (Fig. 4).

### Effect of biochar addition on biochemical analyses

#### Catalase activity

**Fig. 5:**
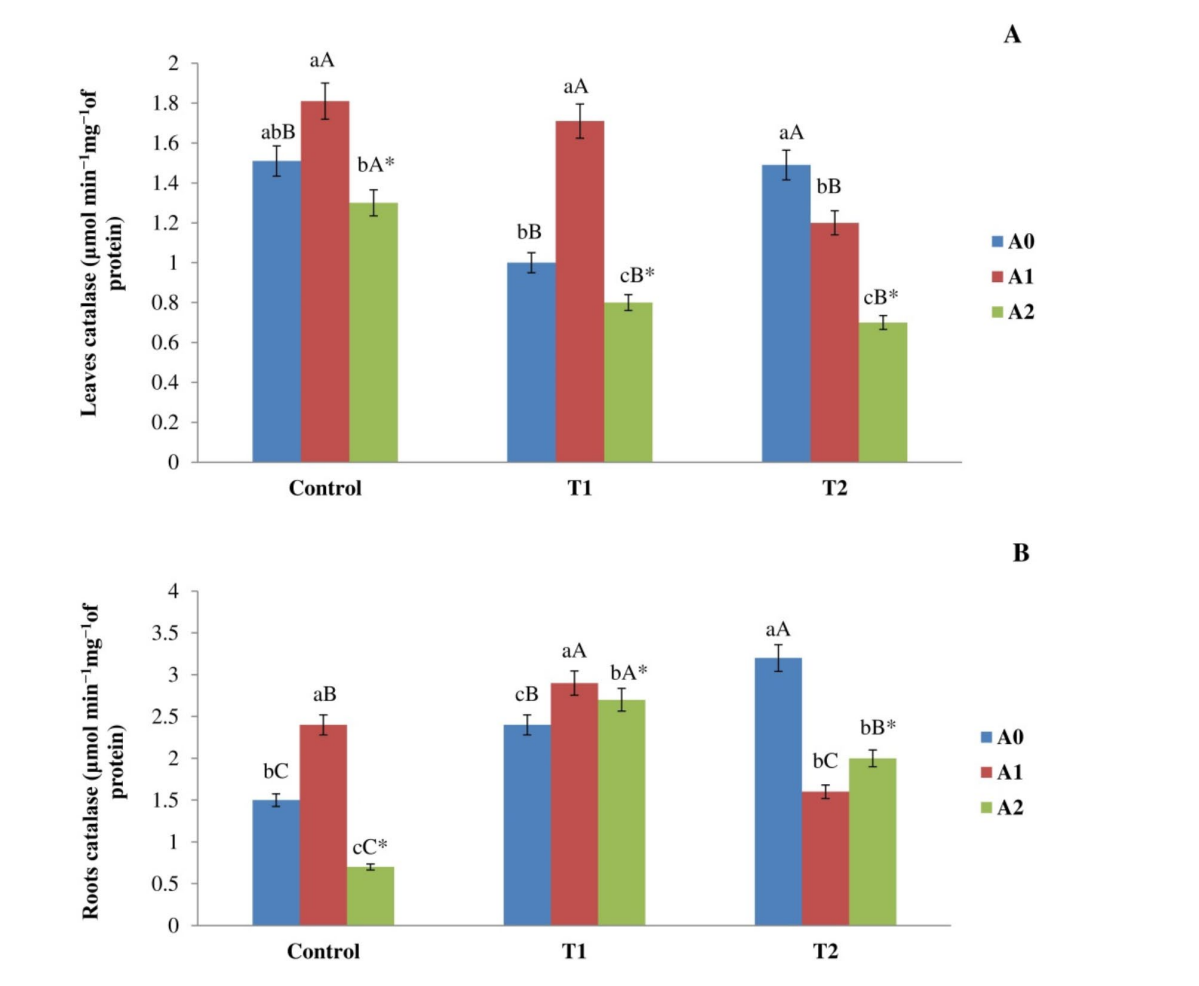
Impact of different levels of salinity (Control, T1, and T2) and rate of biochar (A0, A1, and A2) on the activity of the enzyme catalase in the (**A**) leaves and (**B**) roots of corn plants following 90 days of incubation. The means employed by various capital letters demonstrated a significant disparity in the same biochar addition rate across various treatments. The means employed by various lowercase letters showed a significant disparity in biochar rates within the similar treatment. *: The interactions between biochar rate and salinity have a significant impact.

Figure 5A shows that the control shoots had a high CAT activity, with a value of 1.51 ± 0.06 µmol min^−1^mg^−1^ of protein. The correlation between the level of salinity and the rate of biochar was statistically significant. Plants subjected to A1 demonstrated a notable increase of 19% and 40% in control and T1 compared to the absence of biochar treatment. Nonetheless, the inclusion of A2 led to a decrease in CAT activity in shoots exposed to control soil, T1, and T2, relative to those without biochar treatment. The reduction in CAT activity was 17%, 27%, and 39% for each respective treatment. The addition of biochar to maize roots resulted in a significant rise in CAT activity for T1 and T2. The values recorded were 2.29 ± 0.15 and 3.29 ± 0.88 µmol min^−1^mg^−1^ of protein, respectively, compared to a control group value of 1.49 ± 0.26 µmol min^−1^mg^−1^ of protein. Moreover, the interaction between biochar rate and salt concentration had a substantial effect. The CAT activity of maize roots, cultivated with the inclusion of A1, elevated to an average of 2.4 ± 0.16 and 2.90 ± 0.13 µmol min^−1^ mg^−1^ of protein for the control and T1, respectively. Nonetheless, the integration of A1 led to a 50% decrease in CAT activity in shoots subjected to T2. On the contrary, roots that were administered with A2 exhibited a significant reduction in activity for both the control and T2 treatments than without biochar. The activities were reduced by 55% and 39% respectively (Fig. 5B).

#### Activity of GST

**Fig. 6:**
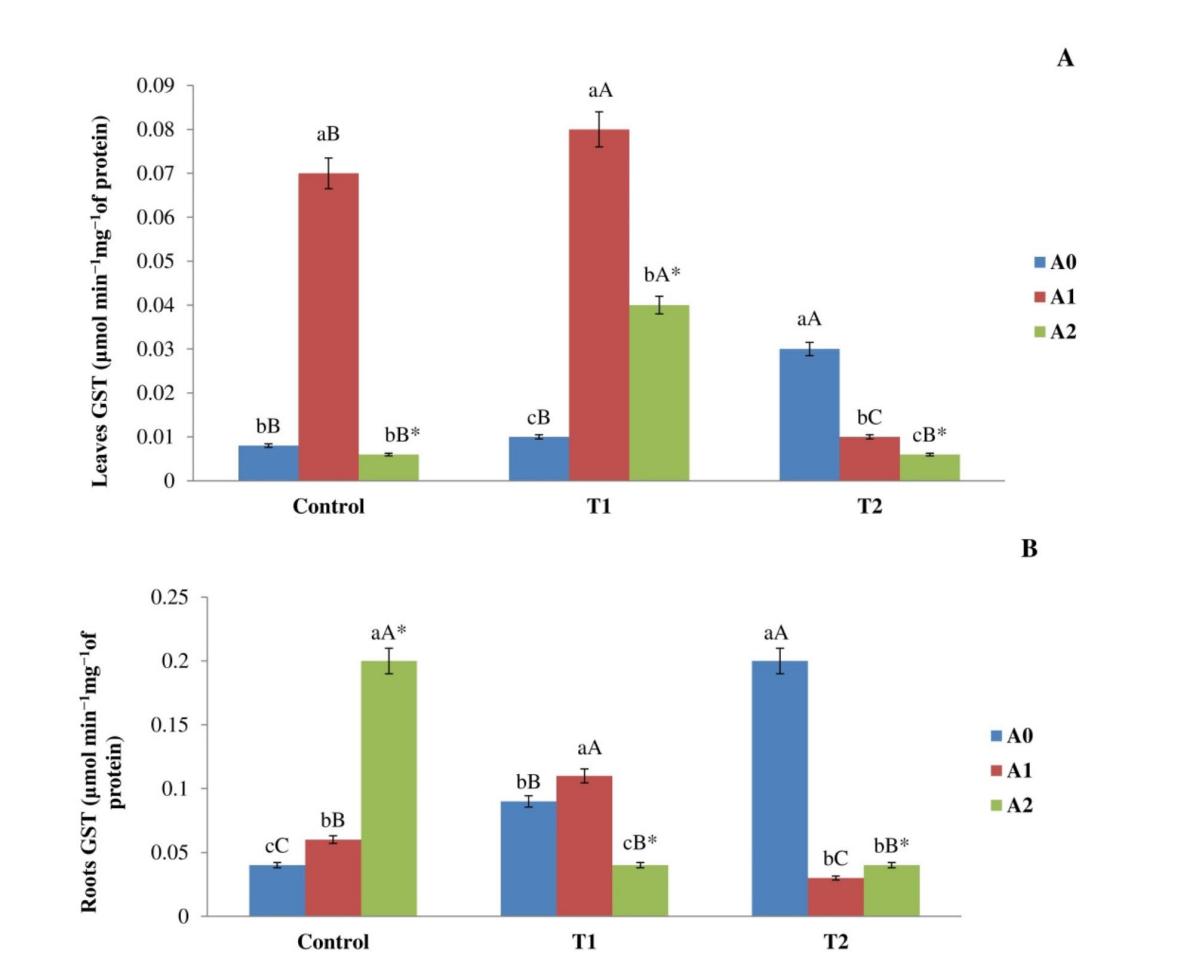
Impact of different levels of salt (Control, T1, and T2) and rate of biochar (A0, A1, and A2) on the activity of GST in the (**A**) leaves and (**B**) roots of corn plants following a 90-day exposure period. The means employed by various capital letters show a significant disparity in the same biochar rate across different treatments. The means employed by various lowercase letters revealed a significant disparity in biochar rates within the similar treatment. *The interactions between biochar rate and salinity have a significant impact.

Figure 6 illustrates the functions of GST in maize plants. The GST activity in shoots subjected to salt (without biochar) was greater than that in the control group. The rise was particularly pronounced in shoots treated with T1, where GST activity attained 0.009 ± 0.0010 µmol min^−1^ mg^−1^ of protein, in contrast to the control group’s value of 0.003 ± 0.0004 µmol min^−1^ mg^−1^ of protein. The correlation between the level of salinity and the rate of biochar was found significant. After being exposed to control and T1 with the addition of A1 for 90 days, the activity of GST showed a significant increase in shoots without the addition of biochar. The average GST activity was 0.07 ± 0.002 and 0.08 ± 0.005 µmol min^−1^ mg^−1^ of protein. However, the GST activity in shoots subjected to T2 with the inclusion of A2 was markedly diminished compared to shoots without biochar, with average values of 0.004 ± 0.0008 µmol min^−1^ mg^−1^ of protein (Fig. 6A). The activity of GST in the roots was increased in T1 and T2 than in control roots. The GST activity was measured as 0.09 ± 0.003 µmol min^−1^mg^−1^ of protein for T1 and 0.20 ± 0.008 µmol min^−1^mg^−1^ of protein for T2, while the control roots had a GST activity of 0.04 ± 0.002 µmol min^−1^mg^−1^ of protein. The correlation between the level of salinity and the rate of biochar was found significant. With the addition of biochar (A1), we noticed a rise in activity of GST for both the control and T1 samples than in roots without biochar. The average GST activity for the control was 0.07 ± 0.002 µmol min^−1^mg^−1^ of protein, whereas for T1 it was 0.2 ± 0.007 µmol min^−1^mg^−1^ of protein. In addition, the presence of A2 led to a significant rise in the activity of GST in the control roots, in comparison to the roots without biochar addition. The resultant value was 0.20 ± 0.015 µmol min^−1^mg^−1^ of protein. Nonetheless, the presence of A2 led to a substantial decrease in GST activity for T1 and T2, in comparison to roots that did not receive the biochar. The measured values were 0.04 ± 0.006 and 0.03 ± 0.004 µmol min^−1^mg^−1^ of protein, respectively (Fig. 6B).

### MDA content concentration in corn plants

**Fig. 7:**
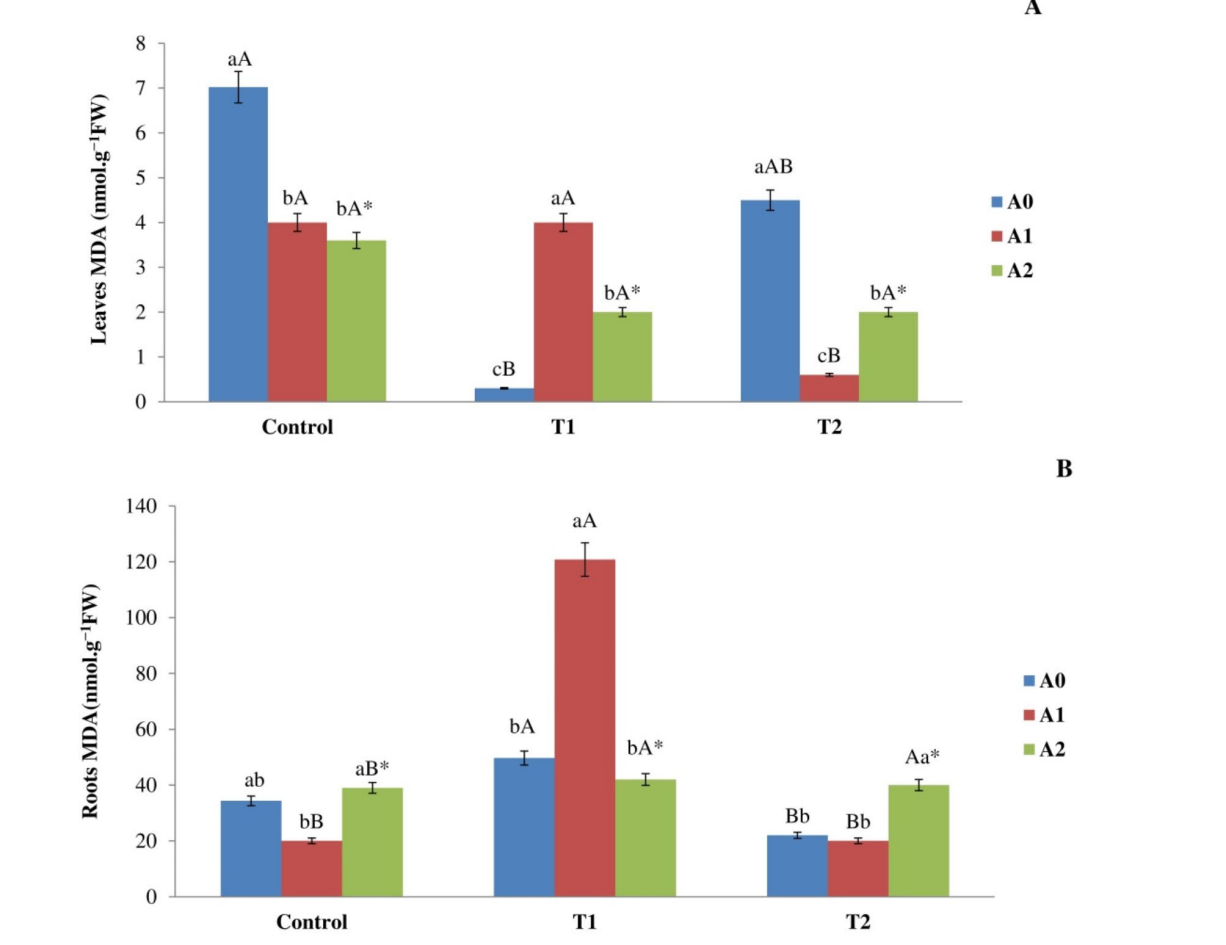
Impact of different levels of salt (Control, T1, and T2) and rate of biochar addition (A0, A1, and A2) on the concentration of MDA in the (**A**) leaves and (**B**) roots of corn plants following a 90-day exposure period. The means employed by various capital letters show a significant disparity for similar biochar addition rates across the various treatments. The means employed by various lowercase letters exhibited a notable disparity in biochar rates within the identical treatment. *: The interactions between biochar rate and salinity have a significant impact.

The concentration of MDA in shoots diminished after 90 days of incubation with escalating salt levels, as illustrated in (Fig. 7). The most notable decrease occurred in plants exposed to T1, registering a value of 0.30 ± 0.06 nmol g^−1^FW, in contrast to the control group’s value of 7.02 ± 0.80 nmol g^−1^FW. The correlation between the level of salinity and the rate of biochar was found significant. The addition of A1 resulted in a reduction of the level of MDA in shoots that were exposed to both the control and T2 (Fig. 7A). This reduction was observed in contrast to shoots that did not add biochar. Specifically, the MDA level was reduced by 40% and 89% in the control and T2. Nonetheless, the inclusion of A1 led to a substantial increase in MDA levels in shoots exposed to both the control and T1 treatments, in contrast to shoots that received biochar. The MDA content increased at a rate of 94%. The A2 amendment resulted in a notable decrease in MDA levels in both control and T2 shoots compared to shoots lacking biochar. The reduction percentages were 45% and 49%, respectively. The corn roots in treatment T1 showed a significant rise in concentration (49.67 ± 7.03 nmol g^−1^FW) than in control roots (34.34 ± 12.79 nmol g^−1^FW). Similarly, the roots in treatment T2 also exhibited a significant increase (30.2 ± 4.72 nmol g^−1^FW) than in control roots (Fig. 7B). The integration of A1 markedly influenced the MDA levels in T1, leading to a substantial increase compared to roots devoid of biochar. The MDA concentration attained a value of 120.80 ± 17.48 nmol g^−1^FW. The addition of A2 markedly increased the MDA concentration in T2 compared to roots devoid of biochar, yielding a value of 50.78 ± 2.28 nmol g^−1^FW.

## Discussion

Addressing soil deterioration resulting from salinity and climate change necessitates the use of innovative strategies to effectively regulate detrimental effects on plant development and improve soil health^2^. Biochar is receiving significant attention for its potential to improve soil quality and fertility, as well as its capacity to immobilize and bioavailability of toxic chemicals^22^. This study aimed to investigate the effects of biochar on the oxidative status and growth parameters of maize plants grown in saline soil. Maize plants exposed to salt stress experienced a significant reduction in both plant dry matter and growth, in comparison to the control plants, was due to soil salinity induces osmotic stress, ion toxicity, low levels of nutrients (nitrogen, calcium, potassium, Phosphorus, iron, Zinc), and oxidative stress in plants, hence restricting the uptake of water from the soil^11^. Wu et al.^23^ assert that salt stress is a primary abiotic factor that limits agricultural productivity. The decline may be attributed to salt’s influence on many metabolic functions and molecular changes^24^. Salt stress markedly affects cell division. Moreover, salt stress in soil adversely affects plant growth due to three variables^25^: Initially, the presence of salt in the soil solution impedes the plant’s ability to absorb water, leading to a decrease in its growth rate. This is a consequence of the osmotic effects induced by salt stress. Secondly, an elevated concentration of sodium ions will increase the osmotic pressure of the soil solution, resulting in a diminished nutrient absorption by plants. Thus, this will have an impact on the availability of essential nutrients of plants such as nitrogen, phosphorus, and potassium. Third, the introduction of excessive salt into the plant’s transpiration stream has a direct impact on leaf the transpiration, resulting in inhibited growth^26^. Therefore, the growth characteristics of corn plants exhibited an increase after the administration of A1 following salt treatment. The incorporation of biochar improves soil fertility, nutrient absorption, and water retention, hence promoting plant development. This may result in enhanced plant development despite the harmful effects of salt^27^. This is attributed to: (i) Biochar’s capacity to diminish soil bulk density and augment total soil porosity, owing to its porous structure and extensive specific surface area, which concurrently fosters root system development, enhances nutrient uptake, and promotes maize growth and yield^27^. Overall, the application of high levels of biochar resulted in a decrease in the growth characteristics of corn plants exposed to T2. Alterations in soil characteristics and indirect release of essential nutrients from applied biochar can cause both increased and decreased plant growth. These changes can also lead to improved uptake of sodium ions without big competition at the surface of roots^28^.

The reduced growth noted under salinity stress conditions may be ascribed to senescence and reduced photosynthetic activity. Thus, a reduction in chlorophyll concentration may also transpire as an additional consequence^2^. In the present study, we observed a reduction in chlorophyll concentration in response to salinity stress, particularly at higher concentrations it was attributed by osmotic stress induced by salt impairs cellular functioning, resulting in cellular damage and a reduction in plant development. Sodium buildup in cells generates secondary stress that adversely impacts key functions, including protein production, photosynthesis, and potassium ion absorption^29^. Similarly, Tauqeer et al.^30^ demonstrated that salinity decreases gas exchange, electron transport chain, and stomatal conductance, and thereby the synthesis of chlorophyll in plants, due to its induction of water and osmotic stress, as well as its harmful effects on plants. Salinity also decreased stomatal conductivity. The Δ consistently decreased with rising external salinity concentration, signifying an enhancement in stomatal restriction of photosynthesis^30^. The decrease in chlorophyll levels can be attributed to the chlorophyll structure degradation caused by the production of reactive oxygen species and the replacement of vital elements with Na^+^ and Cl^−^^31^. Elevated salt concentration will impede the absorption of water and minerals due to the increased extracellular concentration of Na^+^ ions, resulting in heightened osmotic potential. The scarcity of water induces a physiological disruption in plants, resulting in the degradation of chlorophyll levels in the leaves^14^. Nevertheless, the biochar incorporation significantly improved the photosynthetic rate of corn plants, particularly when higher amounts of biochar (A2) were used. This improvement can be attributed to a reduction in Na^+^ level, which subsequently led to an enhancement in the ultrastructure of chloroplast^32^. Biochar can enhance sodium ion absorption in the soil. This method facilitates nutrient release and mitigates osmotic stress by enhancing water retention and augmenting carbon storage. Consequently, there is a substantial enhancement in stomatal conductance, photosynthetic activity, and transcription rates^9^. Chen et al.^33^ also showed that adding biochar to salt-induced wheat plants has a positive impact on decreasing Na-accumulation. The enhanced chlorophyll concentration in corn leaves under higher- salinity, when mixed with biochar, may be attributed to enhanced uptake of carbon dioxide, as demonstrated by^34^. Enhancement in chlorophyll concentratin was attributed due to beneficial impact of biochar supplementation on mitigating sodium buildup in salt-stressed wheat plants. The elevated chlorophyll content in maize leaves under greater salinity conditions with biochar may come from enhanced carbon dioxide uptake^23^.

The negative impact found at the entire plant level resulted in the death of the plant. Salinity impacts crucial plant processes including germination, imbalance of nutrients, and productivity, and induces oxidative stress, was due to elevated saline concentration diminishes osmotic potential, hence constraining germination rate and percentage, as well as root development. It also induces oxidative stress and ion toxicity^35^. Indeed, the elevated levels of salt caused an excessive synthesis of MDA and hydrogen peroxide contents, Salt stress induces increased buildup of reactive oxygen species (ROS), disrupting cellular redox equilibrium and causing oxidative damage, hydrogen peroxide concentration and indicators of oxidative damage to cellular membranes^36,37^. Indeed, several inherent antioxidant enzymes including Glutathione-S-transferase and catalase can remove excessive reactive oxygen species and mitigate their harmful impacts^38^. Production of reactive oxygen species salinity stress in Plants, salt stress disrupts carbon metabolism, thereby promoting the formation of reactive oxygen species (ROS). Stomatal conductance diminishes during salt stress, resulting in reduced transpiration. Stomatal mobility is associated with ion redistribution, alkalization, and ABA buildup^33^. Our findings indicate that lower salt levels in shoots led to a decrease in MDA concentrations in comparison with the control treatments. This could be attributed to the positive impacts of reduced salinity on the physiological processes of plants^39,40^. Lowering salt concentrations can diminish MDA levels, as salt stress elevates lipid peroxidation, resulting in oxidative stress and increased MDA concentrations^32^. However, we observed that the combined application of higher dosages of biochar and salt resulted in a reduction in the MDA concentration in corn leaves than without biochar treatment. Prior research indicated that the use of biochar alleviated salt stress in *Triticum aestivum* by diminishing sodium absorption and enhancing soil ion bioavailability, consequently minimizing their buildup in the plants-^41,42^. In response to elevated salt concentration, root MDA levels were overproduced relative to T1 concentration. Our research indicates that multiple studies indicate that exposure to elevated salt concentrations results in increased MDA levels in maize and wheat compared to plants not subjected to salt stress^43^. Lipid peroxidation can alter mitotic activity, compromise cell membranes, and hence reduce plant growth. Nonetheless, the addition of a reduced amount of biochar led to a decline in MDA concentration relative to roots that did not receive biochar. Numerous investigations have shown that biochar application decreases MDA and hydrogen peroxide concentrations, hence enhancing membrane stability^44^. Our results indicate that MDA levels increase in response to increased stress from salinity and biochar dose. The results suggest that the impact of biochar on salinity may vary according to soil parameters and the quantity of biochar applied^45^. Higher salt levels and biochar additions can enhance membrane breakdown by facilitating the production of reactive oxygen species^46^. Plants have an essential system for reducing reactive oxygen species that protects them from harmful oxidation processes. Antioxidant enzymes are essential in the defence mechanisms against salt within this system^47^. Plant shoots exposed to salinity exhibited a decrease in CAT activity relative to control plants, particularly at T1 concentrations. Haq et al.^48^ found that salinity exposure leads to an overproduction of reactive oxygen species (ROS), subsequently resulting in a decrease in antioxidant enzymes. This may lead to an imbalance between the generation and elimination of reactive oxygen species (ROS). The decline in CAT activity may be ascribed to the detrimental effects of salinity on the enzyme’s production and activation^49^. The activity of CAT in the roots elevated in response to differing salt concentrations, similarly^50^, found that quinoa plants displayed increased catalase activity under salinity stress. This signifies that CAT is essential in converting hydrogen peroxide into oxygen and water. The incorporation of a small quantity of biochar and salt led to an enhancement in CAT activity in both roots and shoots compared to the absence of biochar treatment. Biochar significantly alleviates oxidative stress by inhibiting the production of reactive oxygen species (ROS) and augmenting the activity of antioxidant enzymes in the leaves^4^. The findings suggest that biochar utilization can improve cellular redox equilibrium by augmenting the antioxidant capacity to eliminate reactive oxygen species generated by salt. This subsequently enhances the capacity of plants to withstand salt stress^3^. Additionally, it was found that there were variations in the plant response that had been exposed to salinity to the treatment with biochar. Our findings showed that CAT activity was more significant in the maize roots compared to the shoots when A2 was added, specifically at T1 levels. Biochar can inhibit the movement of sodium ions among various plant tissues. A hypothesis suggests that ion transport in plants may utilize low-affinity cation transporters. According to Kaur et al.^51^, plant roots are considered the primary barrier to salt transport to other tissues. Additionally, the roots shield plant tissues by using antioxidant enzymes. The administration of elevated biochar dosages resulted in reduced catalase activity in the roots and shoots of plants in the T2 treatment compared to untreated plants. The decrease in oxidative stress could be attributed to the reduced accumulation of Na^+^ in plants compared to the control group^52^. Yañez-Yazlle et al.^53^ observed that biochar usage can alleviate the detrimental impacts of salt on plants by altering their stress responses or reducing their exposure to toxic chemicals. To assessed the influence of salinity stress on the oxidative status of maize plants by analyzing GST activity and measuring MDA and CAT levels. Salt stress significantly increased GST activity in both the roots and shoots compared to the control group. Comparable results have been seen for rice and maize under salt stress^54^. GST enzymes facilitate the effective response of plant cells to NaCl toxicity, resulting in the production of MDA^55^. The results of our study indicated that GST activity was elevated in both shoots and roots when subjected to increased levels of biochar. Metwally et al.^56^ discovered that biochar modification improves plant tolerance to salinity stress and alleviates the adverse effects of salinity on plants by increasing antioxidant enzymes. Biochar treatment is essential for modulating enzyme activity and metabolic processes, hence enhancing plant tolerance to salinity stress^57^. Moreover, in the scenario when T1 is present alongside A2, the growth enhancement was more significant in the shoots compared to the roots. The plants have been observed to transport toxic sodium ions into the vacuole through GST enzymes, instead of eliminating them via the roots^58^. Nevertheless, the activity of GST in both roots and shoots exhibited a decline after the application of elevated dosages of biochar to T2. Biochar supplementation might mitigate the detrimental effects of salt by modulating GST activity in plants^1^. The utilization of biochar led to a decrease in Glutathione-S-transferase and ascorbate peroxidase activity in maize plants exposed to salt treatment^59^. Murtaza et al.^60,61^ similarly discovered that the incorporation of biochar into bell bean plants subjected to salinity stress mitigated oxidative damage and elevated GST levels. The findings of our study indicate that biochar significantly mitigates oxidative stress by diminishing the production of hydrogen peroxide and malondialdehyde (MDA). The influence of salinity stress on corn plants was manifested by the reduced activity of antioxidant enzymes following biochar treatment. The growth of maize was evaluated to measure the impacts.

## Conclusion

The results reveal that salinity stress negatively affects biochemical reactions associated with the physiological condition of corn plants, particularly chlorophyll levels. Our findings demonstrated that a dosage of 40 g kg^−1^ biochar can significantly enhance the growth of corn in the presence of high salinity, while also alleviating oxidative stress. This mechanism can be ascribed to a reduction in sodium concentration in plants, potentially resulting from diminished availability in the soil. Nevertheless, higher amounts of biochar application (80 g kg^−1^) did not show efficacy in stimulating plant growth, due to through osmotic stress. And excessive biochar can clog soil pores, which can hinder the exchange of water and nutrients and restrict plant growth. Therefore, it is essential to apply biochar sparingly in saline soils to mitigate salt toxicity and enhance plant growth processes. A comprehensive assessment of biochar application’s impact on carbon sequestration and its potential for climate change mitigation is essential. Biochar has shown efficacy in reducing carbon dioxide emissions, so aiding in the mitigation of climate change. Future research required to check the effect of *acacia*-aged biochar or modified biochar on various crops.

## Data Availability

All the raw data in this research can be obtained from the corresponding authors upon reasonable request.

## Declarations

### Permission

Permissions or licenses were obtained to collect maize variety (SILVER-2019) and *Acacia* bark taken from (Regional Agricultural Research Institute Bahawalpur) before starting the research.

### Statement on guidelines

All experimental studies and experimental materials involved in this research are in full compliance with relevant institutional, national and international guidelines and legislation.
